# CRMP2 Is Involved in Regulation of Mitochondrial Morphology and Motility in Neurons

**DOI:** 10.3390/cells10102781

**Published:** 2021-10-17

**Authors:** Tatiana Brustovetsky, Rajesh Khanna, Nickolay Brustovetsky

**Affiliations:** 1 Department of Pharmacology and Toxicology, Indiana University School of Medicine, Indianapolis, IN 46202, USA; tbrousto@iupui.edu; 2 Department of Pharmacology, College of Medicine, University of Arizona, Tucson, AZ 85721, USA; rkhanna@arizona.edu; 3 Center for Innovation in Brain Sciences, University of Arizona, Tucson, AZ 85721, USA; 4 Stark Neurosciences Research Institute, Indiana University School of Medicine, Indianapolis, IN 46202, USA

**Keywords:** neuron, mitochondria, morphology, motility, CRMP2

## Abstract

Regulation of mitochondrial morphology and motility is critical for neurons, but the exact mechanisms are unclear. Here, we demonstrate that these mechanisms may involve collapsin response mediator protein 2 (CRMP2). CRMP2 is attached to neuronal mitochondria and binds to dynamin-related protein 1 (Drp1), Miro 2, and Kinesin 1 light chain (KLC1). Treating neurons with okadaic acid (OA), an inhibitor of phosphatases PP1 and PP2A, resulted in increased CRMP2 phosphorylation at Thr509/514, Ser522, and Thr555, and augmented Drp1 phosphorylation at Ser616. The CRMP2-binding small molecule (S)-lacosamide ((S)-LCM) prevented an OA-induced increase in CRMP2 phosphorylation at Thr509/514 and Ser522 but not at Thr555, and also failed to alleviate Drp1 phosphorylation. The increased CRMP2 phosphorylation correlated with decreased CRMP2 binding to Drp1, Miro 2, and KLC1. (S)-LCM rescued CRMP2 binding to Drp1 and Miro 2 but not to KLC1. In parallel with CRMP2 hyperphosphorylation, OA increased mitochondrial fission and suppressed mitochondrial traffic. (S)-LCM prevented OA-induced alterations in mitochondrial morphology and motility. Deletion of CRMP2 with a small interfering RNA (siRNA) resulted in increased mitochondrial fission and diminished mitochondrial traffic. Overall, our data suggest that the CRMP2 expression level and phosphorylation state are involved in regulating mitochondrial morphology and motility in neurons.

## 1. Introduction

Adaptive changes in mitochondrial morphology and motility (mitochondrial dynamics) play a crucial role in neuronal responses to fluctuating energy demands in neuronal somata and at nerve terminals [1]. Impairments of mitochondrial dynamics contribute to different neuropathologies, such as Alzheimer’s, Parkinson’s, and Huntington’s diseases [1,2,3]. However, the exact molecular mechanisms involved in regulating mitochondrial dynamics are not completely understood.

Collapsin response mediator proteins (CRMPs) represent a family of cytosolic proteins (CRMP1–5) that are expressed at high levels in the developing brain [4]. CRMPs serve as signaling molecules involved in modulating microtubule polymerization, actin bundling, and endocytosis, resulting in neuronal differentiation. CRMP2 is the most studied among other members of the CRMP family. CRMP2 is a cytosolic phosphoprotein implicated in axon guidance and neurite outgrowth via the Semaphorin 3A pathway [5,6,7]. In contrast to other members of the CRMP family, CRMP2 retains a high level of expression in adults [8]. CRMP2 does not have enzymatic activity and its regulatory actions are mediated by its physical interaction with different proteins [7,9,10], including Kinesin 1 light chain (KLC1) and Dynein, motor proteins involved in axonal transport [11,12].

CRMP2 interaction with other proteins is regulated by CRMP2 post-translational modifications, including phosphorylation [6]. Non-phosphorylated CRMP2 is active and promotes axon/neurite outgrowth, whereas phosphorylated CRMP2 loses its functional activity [13]. CRMP2 can be phosphorylated at different sites by different kinases. Under normal conditions, CRMP2 is phosphorylated up to 30% of maximal phosphorylation [14]. Rho kinase phosphorylates CRMP2 at Thr555 to trigger growth cone collapse [15,16]. In addition, neurite retraction and growth cone collapse are triggered by CRMP2 phosphorylation by glycogen synthase kinase 3β (GSK3β) at Thr509 and Thr514 [13,17]. Cyclin-dependent kinase 5 (Cdk-5) phosphorylates CRMP2 at Ser 522 and primes CRMP2 for subsequent phosphorylation by GSK3β [18,19]. Two phosphatases, protein phosphatase 1 (PP1) and protein phosphatase 2A (PP2A), dephosphorylate CRMP2 at GSK3β sites [20,21]. Inhibiting either phosphatases or kinases may change the CRMP2 phosphorylation state and affect CRMP2′s interaction with other proteins.

CRMP2′s phosphorylation status is reportedly altered in different neuropathologies, including Huntington’s disease (HD) and Alzheimer’s disease (AD). Increased CRMP2 phosphorylation was noted in HD brains [22] and this correlated with excessive mitochondrial fission [23,24,25,26] and reduced mitochondrial motility in HD [25,27,28,29,30], suggesting a mechanistic link between CRMP2 and regulation of mitochondrial dynamics. Similar to HD, CRMP2 hyperphosphorylation [14,31], increased mitochondrial fission, and decreased mitochondrial motility [32,33] were found in AD mouse models, again hinting at a link between the CRMP2 phosphorylation state and alterations in mitochondrial dynamics. However, the extent of CRMP2 involvement in regulating mitochondrial dynamics is still unclear.

CRMP2 binds to mitochondria from neuroblastoma SH-SY5Y cells [34], but its mitochondrial localization and binding partners have not been elucidated. It was reported that CRMP2 binds to mitochondrial adenine nucleotide translocase isoforms 1 and 2 (ANT1 and ANT2) [34], located in the mitochondrial inner membrane (MIM), but whether CRMP2 can interact with other mitochondrial proteins, particularly with proteins involved in regulating mitochondrial dynamics, remains unknown. In the present study, we determined CRMP2 localization in neuronal mitochondria, identified some proteins interacting with CRMP2, and demonstrated CRMP2 involvement in regulating mitochondrial morphology and motility. We also investigated the role of CRMP2 expression and CRMP2 phosphorylation/dephosphorylation in regulating mitochondrial dynamics.

## 2. Materials and Methods

### 2.1. Animals

All procedures with animals were performed in compliance with the US National Institutes of Health Guide for the Care and Use of Laboratory Animals as well as in accordance with the Indiana University School of Medicine Institutional Animal Care and Use Committee approved protocol (# 11385 MD/R). FVB/NJ mice of both sexes were used in this study. Breeding pairs of mice were purchased from Jackson Laboratories (Bar Harbor, ME, USA) and breeding colonies were established in the Laboratory Animals Research Center (LARC) at Indiana University School of Medicine, Indianapolis, IN, USA. The mice were housed under standard conditions with free access to food and water. All mice were housed in polycarbonate cages, 3 mice per cage.

### 2.2. Isolation and Purification of Brain Mitochondria

Percoll gradient-purified brain synaptic (neuronal) mitochondria from FVB/NJ mice were isolated as we previously described [35]. Briefly, brains of three mice (20–22 g weight) were harvested and processed simultaneously. All procedures were performed at 2–4 °C. After homogenization of brain tissue in a 15 mL Dounce homogenizer on ice, 30 mL of Isolation Buffer 1 were added and the diluted homogenate was centrifuged at 2400 rpm for 10 min in a Beckman Avanti J-26XP (Beckman Coulter Life Sciences, Indianapolis, IN, USA) centrifuge, rotor JA 25.50 (700× *g*). After the first centrifugation, the supernatant was centrifuged at 12,500× *g* for 10 min in a Beckman Avanti J-26XP centrifuge, rotor JA 25.50 (18,900× *g*). The pellet, containing synaptosomes, was resuspended in 35 mL of Isolation Buffer 2 and centrifuged at 12,200 rpm for 10 min in a Beckman Avanti J-26XP centrifuge, rotor JA 25.50 (18,900× *g*). The pellet was then resuspended in 5 mL of Isolation Buffer 3 and the suspension was layered onto the top of a discontinuous Percoll gradient (26%/40%) contained within Beckman Ultra-Clear centrifuge tubes. The 26% and 40% Percoll solutions were prepared in Percoll Buffer. The suspension, atop the discontinuous Percoll gradient, was then centrifuged at 15,500× *g* for 28 min in a Beckman Optima L110K ultracentrifuge, bucket rotor SW41Ti (41,100× *g*). Following centrifugation, synaptosomes were collected. In order to obtain synaptic mitochondria, synaptosomes were subjected to nitrogen cavitation using an ice-cold nitrogen cell disruption vessel (Parr Instrument Co., Moline, IL; Cat# 4639), as described previously [35]. Briefly, the synaptosomes were transferred to a 10 mL glass beaker on ice and placed into the nitrogen vessel on ice under 1100 psi (7584 kPa) for 13 min. The ruptured synaptosomes were layered onto a discontinuous Percoll gradient (24%/40%) and centrifuged at 15,500 rpm for 28 min in a Beckman Optima L110K ultracentrifuge, bucket rotor SW41Ti (41,100× *g*). Following centrifugation, synaptic mitochondria were collected and then were washed. Synaptic mitochondria were resuspended in Isolation Buffer 3 and centrifuged at 15,500 rpm for 20 min in a Beckman Optima L110K ultracentrifuge, bucket rotor SW41Ti (41,100× *g*). Mitochondrial pellets were then resuspended in Isolation Buffer 3 and centrifuged again at 15,500 rpm for 20 min in a Beckman Optima L110K ultracentrifuge, bucket rotor SW41Ti (41,100× *g*). The synaptic mitochondria pellet was then resuspended in Isolation Buffer 3 and stored on ice. This was stock suspension of brain synaptic mitochondria. The composition of Isolation Buffer 1: 225 mM mannitol, 75 mM sucrose, 0.1% BSA free from FFA, 10 mM Hepes, pH 7.4 adjusted with KOH, 1 mM EGTA. BSA was used to preserve mitochondrial integrity [36]. The composition of Isolation Buffer 2: 225 mM mannitol, 75 mM sucrose, 10 mM Hepes, pH 7.4 adjusted with KOH, 0.1 mM EGTA. The composition of Isolation Buffer 3: 395 mM sucrose, 0.1 mM EGTA, 10 mM Hepes, pH 7.4. The composition of Percoll Buffer: 320 mM sucrose, 1 mM EGTA, 10 mM Hepes, pH 7.4.

In addition, we purified mouse brain mitochondria using continuous 30% Percoll gradient as it was described recently [37]. Briefly, the crude mixture of synaptic mitochondria was layered on the top of 30% Percoll, prepared in Percoll Buffer, and centrifuged at 33,500 rpm for 30 min in a Beckman Optima L110K ultracentrifuge, fixed-angle rotor 90Ti (95,000× *g*). The resulting mitochondrial pellet was then resuspended in Isolation Buffer 3 and centrifuged again at 15,500 rpm for 20 min in a Beckman Optima L110K ultracentrifuge, bucket rotor SW41Ti (41,100× *g*). The resulting mitochondria pellet was then resuspended in Isolation Buffer 3 and stored on ice. These mitochondria were used for immunoblotting.

### 2.3. Cell Culture

Primary culture of mouse striatal neurons was prepared from postnatal day 1 FVB/NJ mouse pups according to IACUC-approved protocol and procedures published previously [38]. We used neuronal-glial co-cultures derived from postnatal day 1 mouse pups as they are more physiologically relevant and approximate more mature, better developed cells than pure neuronal culture derived from embryonic animals. Based on the ratio of MAP2 (general neuronal marker) and DARPP32 (striatal marker of medium spiny neurons) staining, our cell cultures contained 32.8 ± 7.2% (mean ± SD, *n* = 5 separate platings, Supplementary Materials, Figure S1) of striatal neurons. For fluorescence recordings, neurons were plated on glass bottom Petri dishes as previously described [38]. For all platings, 35 μg/mL uridine plus 15 μg/mL 5-fluoro-2′-deoxyuridine were added 24 h after plating to inhibit proliferation of microglia. Cultures were maintained in a 5% CO_2_ atmosphere at 37 °C in MEM supplemented with 10% NuSerum (BD Bioscience, Bedford, MA, USA), 27 mM glucose.

### 2.4. Immunocytochemistry

Cells were fixed in 4% paraformaldehyde for 15 min. Then, cells were incubated with Protein-Free Blocking Buffer (Pierce, Rockford, IL, USA) for an hour at room temperature. Cells were incubated overnight with primary rabbit anti-CRMP2 antibodies (Sigma, St. Louis, MO, USA, Cat# C2993). Mitochondria were visualized by expressing mitochondrially targeted enhanced yellow fluorescent protein (mito-eYFP, generously provided by Dr. Roger Tsien, UCSD). Then, cells were incubated with secondary donkey anti-rabbit AlexaFluor 568 (Invitrogen, Carlsbad, CA, USA, 1:1000). Bright field and fluorescence images were acquired using a Nikon Eclipse TE2000-U inverted microscope equipped with a Nikon CFI Plan Apo 100× 1.4 NA objective and CCD camera Cool SNAP_HQ_ (Roper Scientific, Tucson, AZ, USA) controlled by MetaMorph, version 6.3 software (Molecular Devices, Downingtown, PA, USA).

### 2.5. Alkali and Trypsin Treatment

For alkali treatment experiments, mouse brain mitochondria (60 µg protein) were incubated on ice at pH 13 (in the standard incubation medium supplemented with 0.1 M Na_2_HCO_3_ plus NaOH as described in [39]) for 0, 15, or 30 min. Then, mitochondrial membranes were pelleted at 35,000 rpm for 30 min in a Beckman Optima L110K ultracentrifuge, fixed-angle rotor 90Ti (105,000× *g*) and used for immunoblotting (20 µg protein per lane). For trypsin treatment experiments, mouse brain mitochondria (80 µg protein) were incubated without or with trypsin (40 µg/mL) for 40 min on ice. At the end of the experiment, trypsin was inhibited by trypsin inhibitor (0.5 mg/mL), mitochondria were treated with Protease Inhibitor Cocktail (Roche, Indianapolis, IN, USA), pelleted as described above, and mitochondrial pellets were taken for immunoblotting analysis (20 µg protein per lane). Where indicated, 10 μg/mL digitonin was used to permeabilize the mitochondrial outer membrane (MOM) as described previously [40].

### 2.6. Immunoblotting

Brain Percoll-purified synaptic mitochondria or mouse striatal neurons cultured on a 60 mm Petri dish were pretreated with Protease Inhibitor Cocktail (Roche) and incubated with NuPAGE LDS sample buffer (Invitrogen, Cat# B0007) plus a reducing agent for 15 min at 70 °C. Bis-Tris gels (4–12%, Invitrogen, Cat# NP0335) were used to separate proteins by electrophoresis (20 µg protein/lane). After electrophoresis, proteins were transferred to a Hybond-ECL nitrocellulose membrane (Amersham Biosciences, Piscataway, NJ, USA, Cat# RPN78D). Blots were incubated at room temperature for 1 h in a blocking solution that was composed of either 5% BSA, phosphate-buffered saline, pH 7.2, plus 0.15% Triton X-100 or 5% milk, phosphate-buffered saline, pH 7.2, plus 0.15% Triton X-100 for total protein immunoblotting, whereas 5% BSA, Tris-HCl buffered saline, pH 7.2, plus 0.15% Triton X-100 was used for phosphoprotein immunoblotting. After blocking, blots were incubated with either rabbit anti-CRMP2 (Sigma, Cat # C2993, 1:1000), rabbit anti-calnexin (Abcam, Cambridge, MA, USA, Cat # ab10286, 1:1200), mouse anti-β-tubulin (Abcam, Cat # 131205, 1:2500), rabbit anti-MEK1/2 (ThermoFisher Scientific, Carlsbad, CA, USA, Cat # MA5-15162, 1:2000), rabbit anti-Histone H3 (ThermoFisher Scientific, Cat # MA5-15150, 1:1000), mouse anti-COX IV (ThermoFisher Scientific, Cat # A21348, 1:2000), mouse anti-Tim23 (BD Biosciences, Franklin Lakes, NJ, USA, Cat # 611222, 1:2500), rabbit anti-VDAC1 (Proteintech, Rosemont, IL, USA, Cat# 10866-1-AP, 1:1500), sheep anti-CRMP2 pThr 509/514 (KinaSource, Dundee, UK, Cat # PB-043, 1:1500), rabbit anti-CRMP2 pSer522 (ECM Biosciences, Versailles, KY, USA, Cat # CP2191, 1:1500), mouse anti-GAPDH (Abcam, Cat # ab9484, 1:2000), rabbit anti-Drp1 pSer 616 (Cell Signaling, Danvers, MA, USA, Cat# 3455, 1:1000), mouse anti-β-actin (ThermoFisher Scientific, Cat# MA5-15739, 1:1000), rabbit anti-Drp1 (Santa Cruz Biotechnology, Paso Robles, CA, USA, Cat# sc-32898, 1:500), rabbit anti-Miro 2 (Proteintech, Cat # 11237-1-AP, 1:1000), or mouse anti-Kinesin 1 light chain (anti-KLC1, EMD Millipore, Cat# MABT 1489, 1:2000) antibodies. Blots were subsequently incubated with either goat anti-mouse or goat anti-rabbit IgG (1:25,000 or 1:20,000, respectively) coupled with horseradish peroxidase (Jackson ImmunoResearch Laboratories, West Grove, PA, USA) and developed with Supersignal West Pico chemiluminescent reagents (Pierce, Rockford, IL, USA, Cat# 32106). Molecular mass marker Page Ruler Plus Prestained Protein Ladder (5 μL, Thermo Fisher; Cat# 26619) was used for molecular mass determination of the bands. The immunoblot images were inverted, and the integrated density of bands was measured after background subtraction using Measurement Long function of Adobe Photoshop 22.2.0.

### 2.7. Co-Immunoprecipitation

Mouse striatal neurons in culture were incubated either without additional treatment, or with 20 nM okadaic acid alone, or with 20 nM okadaic acid plus 30 μM (S)-lacosamide ((S)-LCM) for 16 h. Then cells were lysed in the lysis buffer, containing 139 mM NaCl, 20 mM Tris-HCl, Proteinase Inhibitor Cocktail (Roche), 1% NP40, and 0.1% SDS. Co-immunoprecipitation experiments were performed on freshly prepared cell lysates from mouse striatal neuronal cultures at 12–14 DIV. Lysates were clarified to remove any additional precipitate by incubating with Protein A/G agarose beads (Santa Cruz Biotechnology, Cat# sc-2002, Santa Cruz, CA, USA) for 2 h at 4 °C. Then, the lysates were incubated overnight with primary rabbit anti-CRMP2 (Sigma, Cat # C2993, 1:1000), rabbit anti-Miro 2 (Proteintech, Cat # 11237-1-AP, 1:500), mouse anti-KLC1 (EMD Millipore, Cat# MABT 1489, 1:1000), rabbit anti-Drp1 (Santa Cruz Biotechnology, Cat# sc-32898, 1:100), rabbit anti-Fis1 (ThermoFisher Scientific, Cat# 10956-1-AP, 1:100), mouse anti-Mff (Santa Cruz Biotechnology, Cat# sc-398731, 1:500), rabbit anti-syntaphilin (ThermoFisher Scientific, Cat# 13646-1-AP, 1:500), or rabbit anti-syntabulin (ThermoFisher Scientific, Cat# 16972-1-AP, 1:500) antibodies under gentle agitation at 4 °C followed by incubation with Protein A/G agarose beads (Santa Cruz Biotechnology, Cat# sc-2002) for 2 h at 4 °C. The immune-captured complexes were then washed three times with lysis buffer before being heated at 70 °C in equal volumes of SDS loading dye (Invitrogen, Carlsbad, CA, USA). Samples were then processed by immunoblotting as previously described [41,42]. Blots were probed with rabbit anti-CRMP2, rabbit anti-Drp1, rabbit anti-Miro 2, mouse anti-KLC1, anti-Fis1, rabbit anti-Mff, rabbit anti-syntaphilin, or rabbit anti-syntabulin antibodies (each diluted 1:1000). All blots are representative of at least 3 full cell culture, independent experiments. The immunoblot images were inverted, and Integrated Density of bands was measured after background subtraction using Adobe Photoshop 22.2.0.

### 2.8. Neuronal Transfection

To visualize mitochondria within live cells, cultured striatal neurons were transfected in suspension during plating using an electroporator BTX 630 ECM (Harvard Apparatus, Holliston, MA) with a plasmid encoding mito-eYFP. In some experiments, neurons were co-transfected with both a plasmid encoding mito-eYFP and anti-CRMP2 siRNA (ACTCCTTCCTCGTGTACATTT) [43] or scramble siRNA. In this case, cells expressing mito-eYFP had no detectable CRMP2 (Figure 6A–D). The electroporation procedure usually provided an approximately 10% transfection rate in primary cultures of mouse striatal neurons compared to a <1% efficacy with commercial cationic lipid liposomes. Nevertheless, this transfection rate was not sufficient for immunoblotting or real-time PCR to confirm CRMP2 deletion. Consequently, CRMP2 deletion was analyzed using a single-cell approach. The transfected neurons were imaged 10–12 days after transfection.

### 2.9. Mitochondrial Morphology

Mitochondrial morphology in live cultured striatal neurons was analyzed at room temperature (23 °C) as described previously [44]. Briefly, serial images of neuronal mitochondria visualized with mito-eYFP were collected using spinning-disk confocal microscopy. For this purpose, a Nikon Eclipse TE2000-U inverted microscope equipped with a Yokogawa spinning-disk confocal unit CSU-10, a back-thinned EM-CCD camera Andor iXon^EM^+ DU-897 (Andor Technology, South Windsor, CT, USA), and a motorized flat-top stage Prior H-117 (Prior Scientific, Rockland, MA, USA) was used. This setup was controlled by Andor iQ, version 1.4 software (Andor Technology, South Windsor, CT, USA). To visualize mitochondria, neurons were illuminated at 488 nm using an air-cooled Kr/Ar laser T643-RYB-A02 (Melles Griot, Carlsbad, CA, USA). The laser power was set to the minimal level (<5%), which was sufficient to provide high-quality images and prevent excessive photobleaching. Fluorescence was collected through a 505 nm dichroic mirror and a 535 ± 25 nm emission filter using an objective Nikon CFI Plan Apo 100× 1.4 NA. Serial images (z-stacks) were collected using the piezoelectric positioning device PIFOC^®^ P-721 (Physik Instrumente, Auburn, MA, USA) with a z-step 0.1 μm. While imaging the whole mitochondrial network within neuronal somata, the spatial resolution of the Andor iXon^EM^+ DU-897 camera (pixel size 16 × 16μM) was increased by installing a 2× extender lens in front of the camera. The 3-D blind deconvolution of z-stacks and 3-D rendering was performed using AutoDeblur Gold CF, version 1.4.1 software (MediaCybernetics, Silver Spring, MD, USA). To reconstruct the 3-D structure of neuronal mitochondria, a 3-D maximal projection of the mitochondrial network was created using Imaris, version 5.7.0 software (Bitplane Inc., Saint Paul, MN, USA) as we described previously [44]. To calibrate the image processing and mitochondrial measurements, fluorescent microbeads were used [44]. The length of mitochondria was measured with individual mitochondria located in neuronal processes. For each experimental condition, 100 randomly chosen mitochondria from at least 10 neurons from three different platings were analyzed. During fluorescence measurements, neurons were incubated in the standard bath solution containing 139 mM NaCl, 3 mM KCl, 0.8 mM MgCl_2_, 1.8 mM CaCl_2_, 10 mM NaHEPES, pH 7.4, 5 mM glucose, and 65 mM sucrose. Sucrose was used to maintain osmolarity similar to that in the growth medium (340 mosm). The osmolarity of the solutions was measured with the osmometer Osmette II™ (Precision Systems Inc., Natick, MA, USA).

### 2.10. Mitochondrial Motility

Mitochondrial motility in striatal cultured neurons was assessed at 37 °C using wide-field fluorescence microscopy. Mitochondrial traffic was recorded with a Nikon Eclipse TE2000-U inverted microscope using a Nikon objective Nikon CFI Plan Apo 100× 1.4 NA and Photometrics Cool SNAP_HQ_ camera (Roper Scientific, Tucson, AZ, USA) controlled by MetaMorph, version 6.3 software (Molecular Devices, Downingtown, PA, USA). The excitation light (480 ± 20 nm) was delivered by a Lambda-LS system (Sutter Instruments, Novato, CA, USA) and fluorescence was measured through a 505 nm dichroic mirror at 535 ± 25 nm. The images were acquired during the time-course of the experiment (5 min) with a frequency of 1 Hz. The motility of neuronal mitochondria was analyzed after constructing kymographs using NIH ImageJ, version 1.53a software.

### 2.11. Statistics

Data are displayed as the mean ± SD of the indicated number of separate experiments. Statistical analysis of the experimental results consisted of an unpaired *t*-test or one-way analysis of variance (ANOVA) followed by Bonferroni post hoc test (GraphPad Prism^®^ version 4.0, GraphPad Software Inc., La Jolla, CA, USA). Every experiment was performed using several different preparations of isolated mitochondria or cultured neurons.

## 3. Results

### 3.1. CRMP2 Localization on Mitochondria

**Figure 1:**
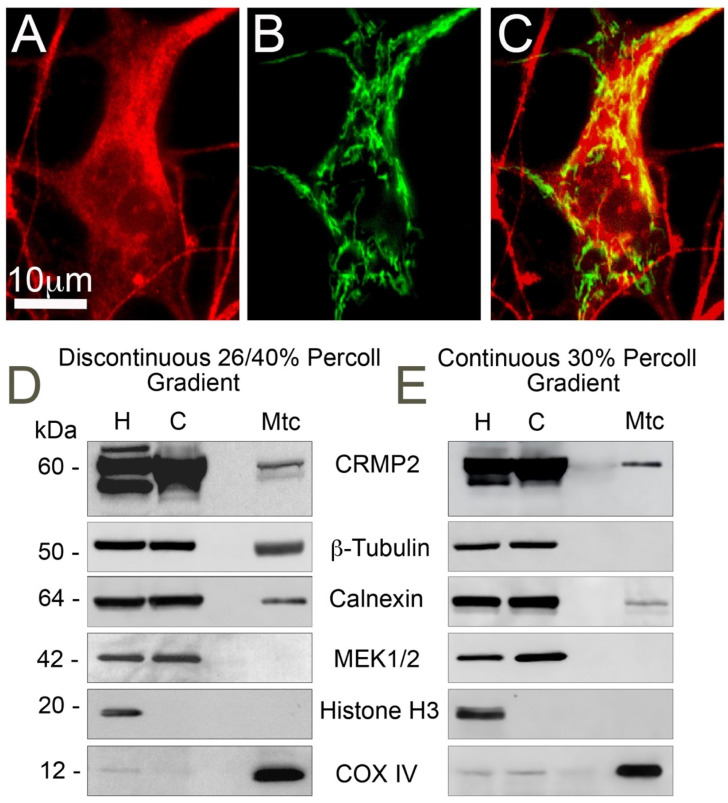
Interaction of CRMP2 with brain mitochondria. (**A**–**C**) Immunocytochemistry staining with anti-CRMP2 antibody (**A**, red) and visualization of mitochondria with mitochondrially targeted enhanced yellow fluorescent protein (mito-eYFP) (**B**, green) illustrates co-localization of CRMP2 and mitochondria (yellow, **C**) in cultured striatal neuron from FVB/NJ mouse. (**D**,**E**), CRMP2 binds to synaptic brain mitochondria isolated from FVB/NJ mice. Mitochondria were purified either on discontinuous 26/40% Percoll gradient (**D**) or on continuous 30% Percoll gradient (**E**) as described in the Materials and Methods. H: homogenate; C: cytosolic fraction; Mtc: mitochondrial fraction. MEK1/2, calnexin, β-tubulin, Histone H3, and COX IV are cytosolic, ER, microtubule, nuclear, and mitochondrial markers, respectively. Representative western blots from 3 independent experiments are shown.

CRMP2 is an abundant cytosolic phosphoprotein [5,6,7], which surrounds mitochondria in the cell. It has been previously shown that CRMP2 binds to mitochondria from neuroblastoma SH-SY5Y cells [34]. In immunocytochemistry experiments, we found that mitochondria co-localize with CRMP2 in mouse cultured striatal neurons (Figure 1A–C) with Pearson’s correlation coefficient r = 0.547 ± 0.042 (mean ± SD, *n* = 5, calculated with NIH ImageJ 1.53k software, JACoP plugin). This suggested that CRMP2 may interact with mitochondria in neurons. Indeed, in immunoblotting experiments, we found CRMP2 protein in a brain mitochondria fraction purified on discontinuous 26/40% Percoll gradient (Figure 1D). However, in this fraction, we also found calnexin and β-tubulin, endoplasmic reticulum (ER), and microtubule markers, respectively, indicating ER and microtubule association with mitochondria. To better purify brain mitochondria, we utilized continuous 30% Percoll gradient as described recently [37]. This procedure decreased the β-tubulin level below the detection limit of immunoblotting but failed to eliminate CRMP2 and calnexin (Figure 1E). This suggests that CRMP2 binds to brain mitochondria and is not a contaminant associated with β-tubulin, which is known to bind to CRMP2 [45].

**Figure 2:**
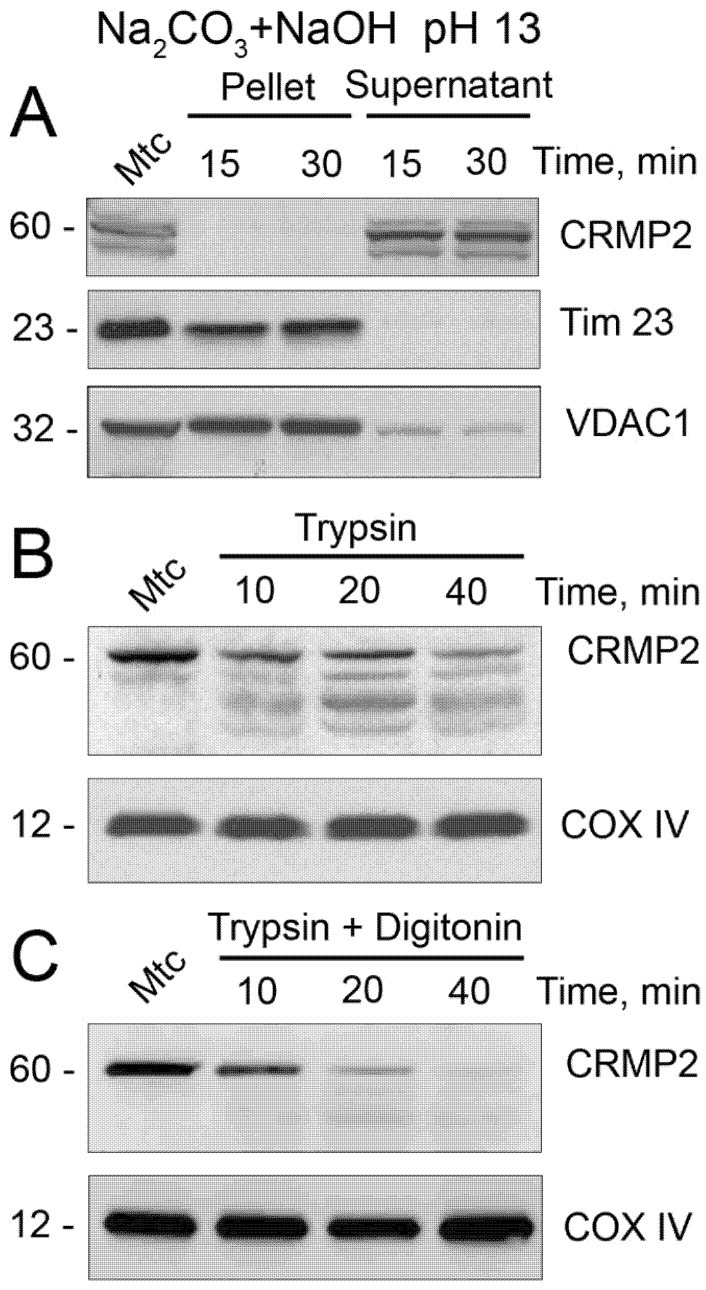
CRMP2 localization on mitochondria. (**A**) Alkali treatment removes CRMP2 from mitochondrial membranes, suggesting that CRMP2 is not embedded in the mitochondrial membranes. In (**A**), brain synaptic mitochondria were incubated on ice at 0 °C for the indicated time with 0.1 M Na_2_CO_3_, pH was adjusted to 13 with NaOH. Then, mitochondrial membranes were pelleted and CRMP2 presence in the pellets and supernatants was evaluated using immunoblotting. Tim 23 is a marker for the mitochondrial inner membrane; VDAC1 is a marker for the mitochondrial outer membrane. (**B**,**C**) Trypsin treatment degrades CRMP2 bound to purified synaptic brain mitochondria from FVB/NJ mice (**B**). In (**B**), mitochondria were incubated on ice at 0 °C for the indicated time with 40 µg/mL trypsin. In (**C**), mitochondria were incubated on ice at 0 °C for the indicated time with 40 µg/mL trypsin plus 10 µg/mL digitonin. Without trypsin, CRMP2 was not degraded. Cytochrome oxidase subunit IV (COX IV) is a loading control. Mtc, mitochondria. Representative Western blots from 3 independent experiments are shown.

Alkali treatment (pH 13) of isolated brain synaptic (neuronal) mitochondria for 15 min completely washed out CRMP2, indicating that CRMP2 is not embedded into mitochondrial membranes (Figure 2A). Treating isolated brain synaptic mitochondria with 40 μg/mL trypsin for 40 min at room temperature (23 °C) significantly, but not completely, degraded CRMP2, suggesting that most CRMP2 is attached to the outer side of the mitochondrial outer membrane (MOM, Figure 2B). Treating mitochondria with 10 μg/mL digitonin to permeabilize the MOM resulted in complete degradation of CRMP2 by trypsin (Figure 2C), suggesting that some CRMP2 could be located behind the MOM in the intermembrane space.

### 3.2. Manipulations with CRMP2 Phosphorylation

**Figure 3:**
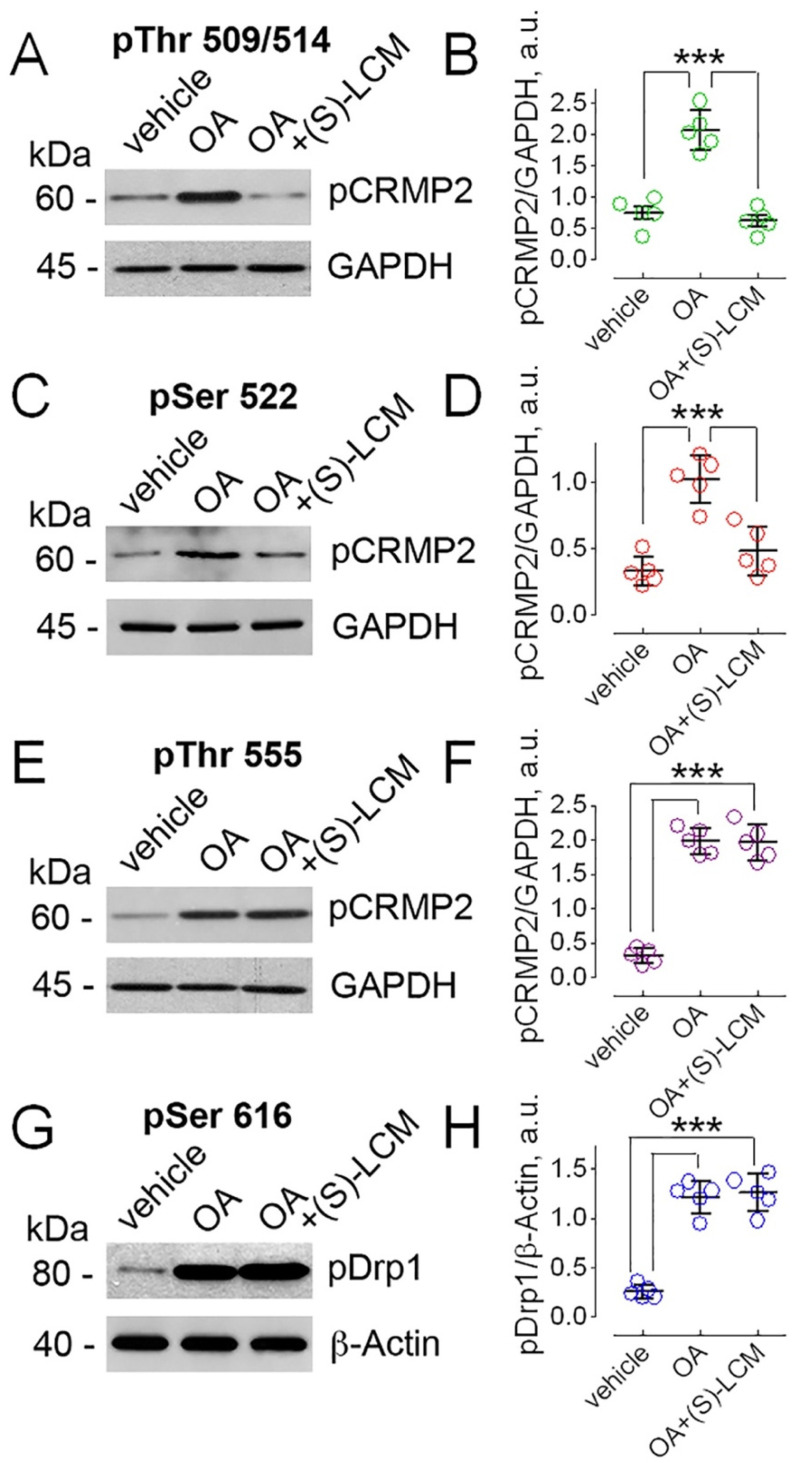
Inhibition of PP1 and PP2A in mouse striatal neurons treated with okadaic acid (OA) increased CRMP2 phosphorylation at Thr509/514, Ser522, and Thr555, and increased Drp1 phosphorylation at Ser616. (S)-lacosamide ((S)-LCM) prevented CRMP2 phosphorylation at Thr509/514 and Ser522 but not at Thr555 and Drp1 phosphorylation at Ser616. Neurons were treated with 20 nM OA and/or 30 µM (S)-LCM for 16 h as indicated. 0.01% DMSO was used as a vehicle. GAPDH and β-actin are loading controls. In (**A**,**C**,**E**,**G**), representative Western blots are shown. In (**B**,**D**,**F**,**H**) pCRMP2 or pDrp1 densitometry data, normalized per GAPDH or β-actin, are shown. Data are mean ± SD. The colored circles indicate data from individual measurements. In (**B**,**D)**, *** *p* < 0.001 comparing vehicle with OA and OA with OA plus (S)-LCM, *n* = 5 separate, independent experiments. In (**F**,**H**), *** *p* < 0.001 comparing vehicle with OA and OA plus (S)-LCM, *n* = 5 separate, independent experiments.

CRMP2 expression and localization can be modified by post-translational modifications, including phosphorylation [6,10]. Therefore, next we tested if we could manipulate the CRMP2 phosphorylation state. For these studies, we used mouse striatal neurons in culture, a model system we have used previously to investigate the mechanisms contributing to the pathogenesis of Huntington’s disease [46,47]. In mouse cultured striatal neurons, inhibiting phosphatases PP1 and PP2A for 16 h with 20 nM okadaic acid (OA, Abcam, Cambridge, MA, USA), a potent PP1 and PP2A inhibitor [48,49], resulted in increased CRMP2 phosphorylation at Thr509/514 (Figure 3A,B), Ser522 (Figure 3C,D), and Thr555 (Figure 3E,F). Similar results were observed previously in primary cultures of rat cortical neurons [50]. In addition to CRMP2, OA also increased the phosphorylation of Drp1 at Ser616 (Figure 3G,H), in line with previously reported data [51]. (S)-lacosamide ((S)-LCM, 30 μM)), a small molecule that specifically binds to CRMP2 [52], prevented CRMP2 phosphorylation at Thr509/514 and Ser522 (Figure 3A–D), consistent with previously published results [53], but did not influence CRMP2 phosphorylation at Thr555 and Drp1 phosphorylation at Ser616 (Figure 3E–H).

### 3.3. CRMP2 and Proteins Involved in Regulating Mitochondrial Morphology and Motility

**Figure 4:**
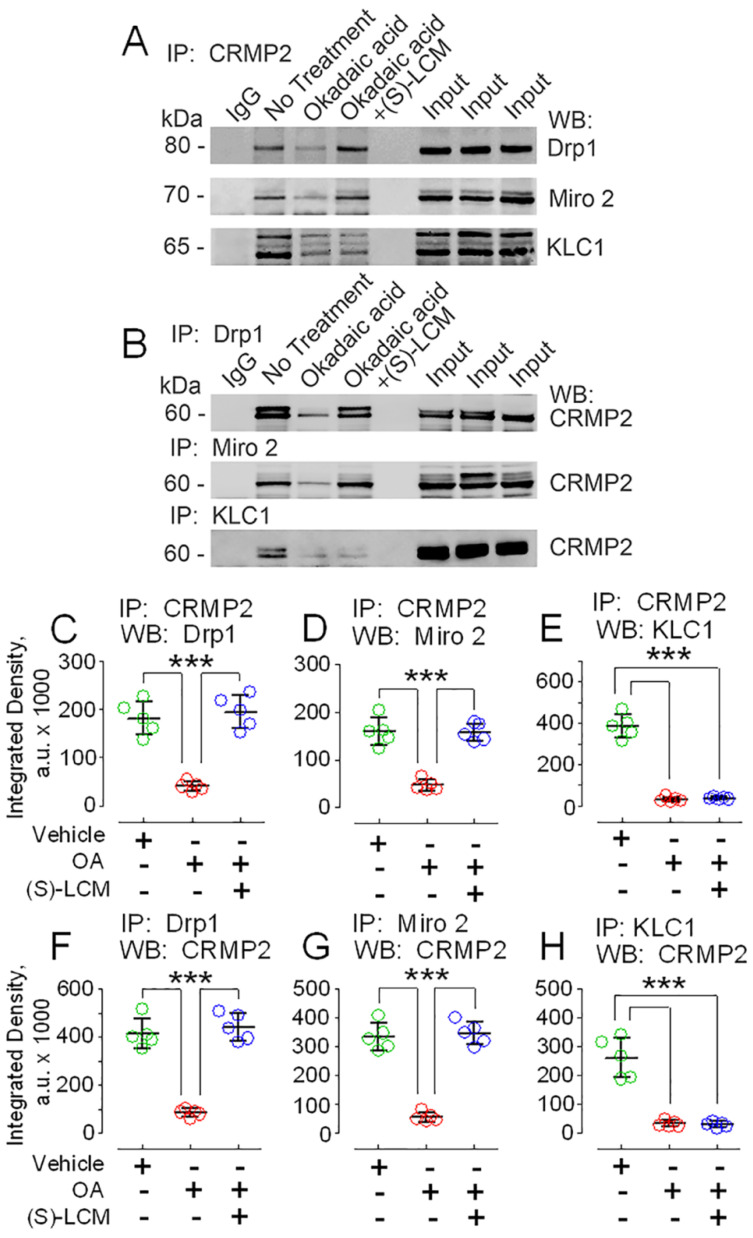
CRMP2 co-immunoprecipitated with Drp1, Miro 2, and Kinesin 1 light chain (KLC1) in lysates of mouse cultured striatal neurons. Incubation of neurons with okadaic acid (OA) disrupted CRMP2 binding to Drp1, Miro 2, and KLC1. (S)-lacosamide ((S)-LCM) prevented CRMP2 dissociation from Drp1 and Miro 2 but not from KLC1. In (**A**), immunoprecipitation (IP) with anti-CRMP2 antibody followed by Western blotting (WB) with anti-Drp1, anti-Miro 2, and anti-Kinesin 1 light chain (KLC1) antibodies, In (**B**), IP with anti-Drp1, anti-Miro 2, and anti-KLC1 antibodies followed by WB with anti-CRMP2 antibody. Neurons were treated with 20 nM OA alone or with 20 nM OA and 30µM (S)-LCM for 16 h. Here, 0.01% DMSO was used as a vehicle. The input was 5% of total protein used in the pull-down procedure. In (**C**–**H**), densitometry data are shown. The colored circles indicate data from individual measurements. Data are mean ± SD. *** *p* < 0.001 comparing vehicle with OA or OA plus (S)-LCM, *n* = 5 separate, independent experiments.

The functional consequences of CRMP2 association with mitochondria and the role of CRMP2 phosphorylation in CRMP2 binding to mitochondrial targets are not completely clear. Because most CRMP2 binds to the outer side of the MOM, we hypothesized that CRMP2 could be involved in regulating mitochondrial morphology and/or motility, two major components of mitochondrial dynamics that could be modulated from outside of the mitochondria. Co-immunoprecipitation (co-IP) experiments with mouse cultured striatal neurons revealed that CRMP2 interacts with Drp1 and Miro 2 (Figure 4A–D,F,G), proteins involved in mitochondrial fission and motility, respectively [1]. At the same time, we did not find evidence for CRMP2 binding to mitochondrial outer membrane proteins—mitochondrial fission protein 1 (Fis1), mitochondrial fission factor (Mff), the mitochondrial trafficking protein syntaphilin, and the mitochondrial docking protein syntabulin (not shown). Recently, Mokhtar et al. reported that CRMP2 interacts with KLC1 and CRMP2 phosphorylation at Thr555 negatively correlated with CRMP2-KLC1 interaction [22]. In our experiments, we also found CRMP2 interaction with KLC1 (Figure 4A,B,E,H). OA (20 nM, incubation with neurons for 16 h) significantly decreased interaction of CRMP2 with Drp1, Miro 2, and KLC1 (Figure 4A–H). On the other hand, (S)-LCM (30 μM, incubation with neurons for 16 h) prevented the OA-induced decrease in CRMP2 binding to Drp1 and Miro 2 but not to KLC1 (Figure 4A–H), in parallel with changes or lack thereof in CRMP2 phosphorylation (Figure 3A–F). These observations suggest that CRMP2 binding to Drp1 and Miro 2, but not to KLC1, could be regulated by CRMP2 phosphorylation at Thr509/514 and/or Ser522. CRMP2 binding to proteins participating in regulation of mitochondrial morphology and motility suggests that CRMP2 might be involved in modulating mitochondrial dynamics.

### 3.4. CRMP2 Phosphorylation State and Alterations in Mitochondrial Morphology and Motility

**Figure 5:**
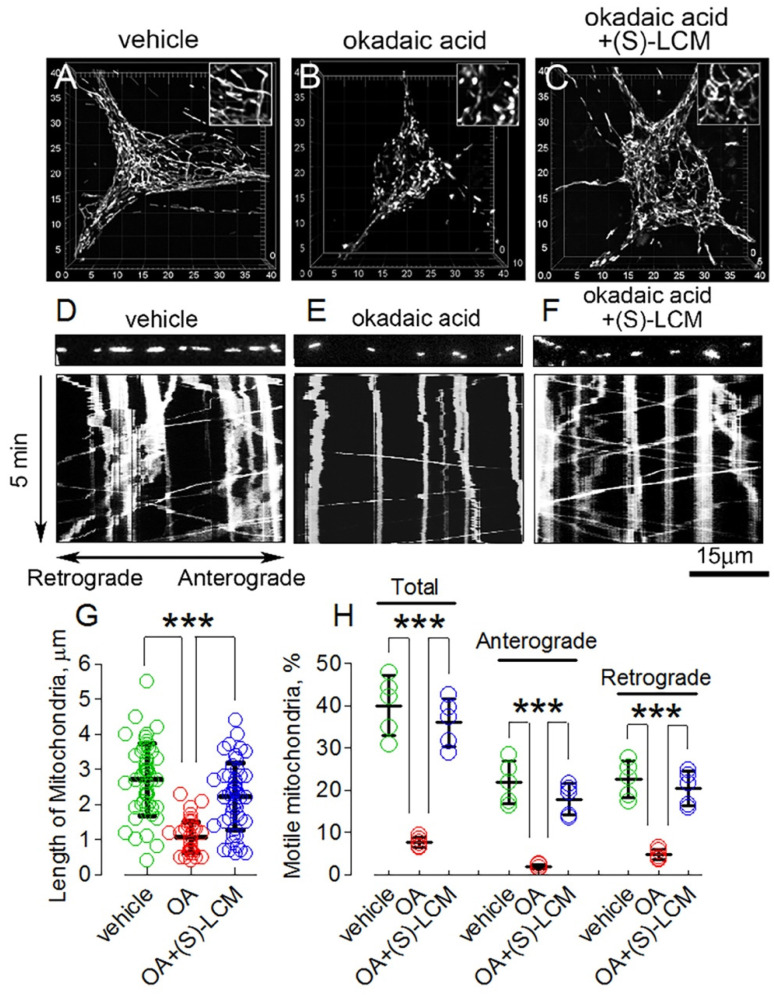
The okadaic acid (OA) induced an increase in mitochondrial fission and suppressed mitochondrial motility in cultured striatal neurons (10 DIV). (S)-lacosamide ((S)-LCM) rescued mitochondrial morphology and motility. Mitochondria were visualized by expressing mitochondrially targeted enhanced yellow fluorescent protein (mito-eYFP). Serial images (z-stacks) were collected as described in Materials and Methods. In (**A**), a 3-D image shows normal mitochondria in neuron treated with 0.01% DMSO, a vehicle. In (**B**), increased fission in neuron treated with 20 nM OA for 16 h. In (**C**), preservation of mitochondrial morphology in neuron treated with 30 µM (S)-LCM and 20 nM OA for 16 h. In Insets, mitochondria are shown at additional 2x magnification. In (**D**–**E**), mitochondrial motility was recorded at 37 °C for 5 min, and kymographs were constructed using NIH ImageJ 1.53a software. The angled traces indicate moving mitochondria, the straight vertical traces indicate stationary mitochondria. The strips show fluorescent images of mitochondria at the start of recordings. Where indicated, the OA (20 nM OA for 16 h) inhibited mitochondrial traffic in cultured striatal neurons (**E**). In (**F**), (S)-LCM (30 µM (S)-LCM for 16 h) rescued mitochondrial motility. In (**D**), 0.01% DMSO was used as a vehicle. In (**G**), the length of mitochondria is in μm. In total, 100 randomly chosen mitochondria from at least 10 neurons from three different platings were analyzed. Data are mean ± SD. In (**H**), the percentages of total motile mitochondria and mitochondria moving in anterograde and retrograde directions are shown. Data are mean ± SD. In (**G**,**H**), *** *p* < 0.001 comparing OA with vehicle and OA plus (S)-LCM, *n* = 5 separate, independent experiments. The colored circles indicate data from individual measurements.

OA induced CRMP2 hyperphosphorylation at Thr509/514, Ser522, and Thr555 (Figure 3A–F) and decreased the interaction of CRMP2 with Drp1, Miro 2, and KLC1 (Figure 4) correlated with shortening of mitochondria (Figure 5A,B,G), indicative of augmented fission, and suppressed mitochondrial traffic (Figure 5D,E,H). (S)-LCM decreased CRMP2 phosphorylation at Thr509/514 and Ser522 but not at Thr555 (Figure 3A–F), restored interaction of CRMP2 with Drp1 and Miro 2 but not with KLC1 (Figure 4), and rescued mitochondrial morphology (Figure 5C,G) and motility (Figure 5F,H) in mouse cultured striatal neurons treated with 20 nM OA for 16 h. Without OA, (S)-LCM was ineffective (not shown).

### 3.5. CRMP2 Deletion Correlates with Alterations in Mitochondrial Morphology and Motility

**Figure 6:**
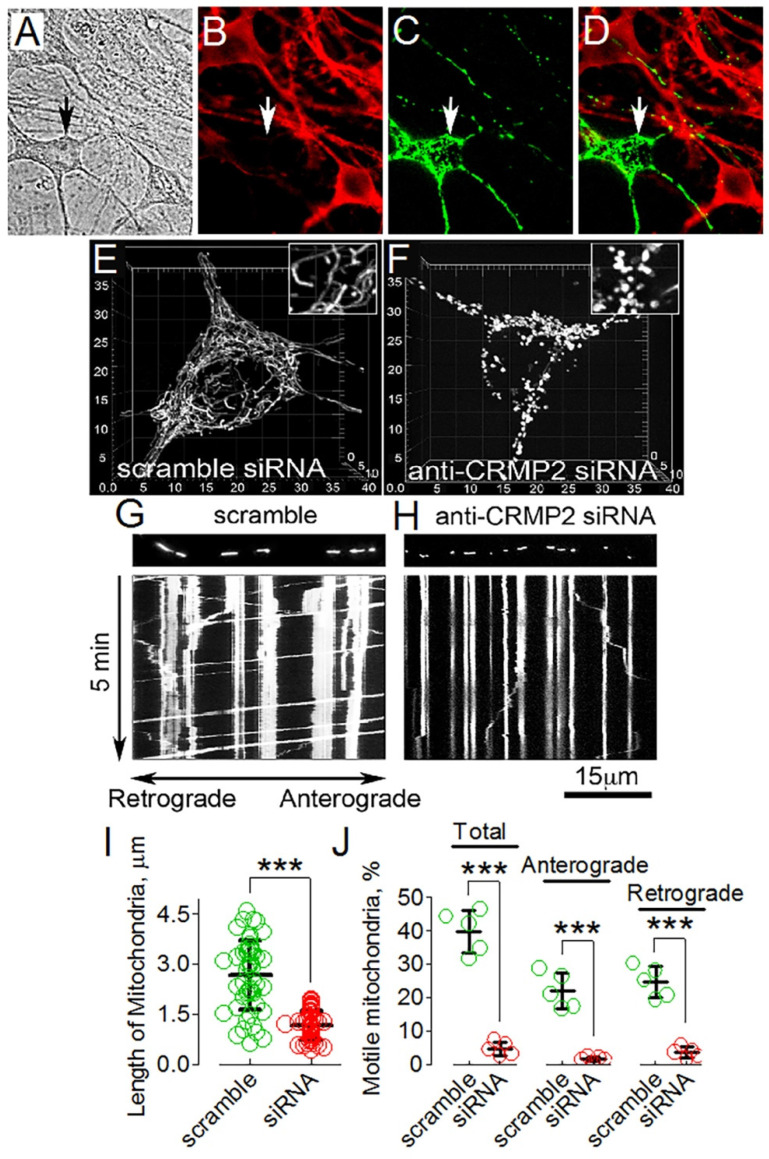
CRMP2 deletion with siRNA (ACTCCTTCCTCGTGTACATTT) increased mitochondrial fission and suppressed mitochondrial motility in cultured striatal neurons (10 DIV). In (**A**–**D**), a representative immunochemistry experiment is shown. In (**A**), bright-field image. (**B**), CRMP2 staining. (**C**), mitochondrial staining with mito-eYFP. (**D**), CRMP2 and mitochondria staining overlay. *Arrows*, transfected neuron. In (**E**–**H**), results of representative experiments with live cultured striatal neurons are shown. In (**E**,**F**,**I**), CRMP2 deletion correlated with increased fission of mitochondria in cultured striatal neurons. Mitochondria were visualized in live neurons by mito-eYFP. Mitochondrial morphology was analyzed using 3-D reconstruction of serial images (z-stacks), which were collected as described in Materials and Methods. In (**E**), neurons were transfected with scramble siRNA; mitochondria remained normal. Transfection with anti-CRMP2 siRNA led to deletion of CRMP2 (**A**–**D**) and mitochondrial shortening (**F**,**I**), suggesting excessive mitochondrial fission. In (**I**), the length of mitochondria in μm. In total, 100 randomly chosen mitochondria from at least 10 neurons from three different platings were analyzed. *** *p* < 0.001 comparing scramble with anti-CRMP2 siRNA. Data are mean ± SD. In (**G**,**H**,**J**), CRMP2 deletion with siRNA correlated with inhibition of mitochondrial traffic in live cultured striatal neurons. In G, mitochondrial traffic in a neuron transfected with scramble siRNA. (**H**), mitochondrial traffic in a neuron transfected with anti-CRMP2 siRNA. Vertical traces show stationary mitochondria, angled traces indicate moving organelles. The strips show fluorescent images of mitochondria at the start of recordings. Mitochondrial motility was recorded for 5 min at 37 °C. In (**J**), the percentages of motile mitochondria and mitochondria moving in the anterograde and retrograde directions are shown. *** *p* < 0.001 comparing scramble with anti-CRMP2 siRNA. Data are mean ± SD, *n* = 5 separate, independent experiments. The colored circles indicate data from individual measurements.

To test whether alterations in mitochondrial dynamics following pharmacological interventions could indeed be due to CRMP2, we investigated the effect of genetic deletion of CRMP2 on mitochondrial morphology and motility. We downregulated CRMP2 in mouse cultured striatal neurons using anti-CRMP2 siRNA delivered by electroporation (Figure 6A–D). Simultaneously, neurons were transfected with cDNA encoding mitochondrially targeted enhanced yellow fluorescent protein (mito-eYFP) to visualize mitochondria in transfected cultured neurons. CRMP2 was strongly downregulated in transfected neurons, which could be easily identified by mito-eYFP fluorescence. These experiments were performed with fixed cells using immunocytochemistry (Figure 6A–D). In experiments with live cultured striatal neurons with downregulated CRMP2, identified by mito-eYFP fluorescence, we found that CRMP2 downregulation correlated with shortening of mitochondria, indicating increased mitochondrial fission (Figure 6E,F,I). Additionally, mitochondrial motility in these cells was suppressed (Figure 6G,H,J). These findings supported our hypothesis regarding CRMP2 involvement in regulating mitochondrial dynamics. The results obtained with genetic deletion of CRMP2 recapitulated alterations in mitochondrial morphology and motility induced by CRMP2 hyperphosphorylation in the presence of OA (Figure 5). However, (S)-LCM was ineffective and could not rescue mitochondrial morphology and motility in neurons with downregulated CRMP2 (not shown). Overall, our results strongly suggest that CRMP2′s expression level as well as its phosphorylation state are involved in regulating the morphology and motility of neuronal mitochondria.

## 4. Discussion

CRMP2 is a cytosolic phosphoprotein that interacts with different proteins, modulating their activity and/or location [5,6,7]. The cytosolic provenance of CRMP2 favors its interaction with different organelles, including mitochondria. It has been shown that CRMP2 binds to mitochondria in neuroblastoma SH-SY5Y cells [34]. In our experiments, we found that CRMP2 co-localizes with mitochondria in mouse cultured striatal neurons and is present in the fraction of isolated, highly purified brain mitochondria, suggesting CRMP2 binding to these organelles. Importantly, manipulations with CRMP2 expression level or phosphorylation status overtly influenced mitochondrial morphology and motility, supporting direct interaction of CRMP2 with mitochondria.

In our experiments, CRMP2 was completely removed from mitochondria by alkali treatment, indicating that CRMP2 is not embedded into mitochondrial membranes. Interestingly, CRMP5, another member of the CRMP family, was found in the mitochondrial inner membrane (MIM) and appeared to be inserted into mitochondrial membranes [54]. In our experiments, we found that CRMP2 was significantly, but not completely, degraded following a 40-min incubation of mitochondria with trypsin. This raises the possibility that at least a fraction of CRMP2 could be internalized into mitochondria and could interact with the MIM. However, there is no evidence that CRMP2 has a mitochondrial leader sequence, and therefore, it is unlikely to cross the MOM and enter mitochondria. There is some evidence that during tissue homogenization and mitochondria isolation procedures, mitochondria could entrap some extramitochondrial components, leading to artifact [55]. This could explain the presence of the remaining CRMP2 in the mitochondrial fraction following treatment with trypsin. Consequently, we posit that CRMP2 is most likely localized on the cytosolic side of the MOM.

CRMP2 interactions with its binding partners can modulate their activity and/or interaction with other proteins. CRMP2 interaction with other proteins is modulated by CRMP2 post-translational modifications, including phosphorylation [6]. CRMP2 phosphorylation decreases its interaction with different proteins [56] and may lead to significant consequences for neurons [6]. Under normal conditions, CRMP2 is phosphorylated up to 30% of the maximal phosphorylation [14]. Inhibiting either phosphatases or kinases may change the CRMP2 phosphorylation state, and thus impact CRMP2 interactions with other proteins [56]. CRMP2 can be phosphorylated at multiple sites by different kinases. Protein kinase Cdk-5 phosphorylates CRMP2 at Ser522 and predisposes CRMP2 for subsequent phosphorylation by GSK-3β at Thr509 and Thr514 [18,19]. Because phosphorylation of Ser522 by Cdk5 primes CRMP2 phosphorylation at GSK-3β sites [18,19], inhibiting Cdk5-mediated phosphorylation of CRMP2 suppresses phosphorylation of GSK-3β sites. (S)-LCM binds to CRMP2 [52] and specifically inhibits Cdk-5-mediated CRMP2 phosphorylation at Ser522 [53], and subsequently suppresses GSK3β-mediated CRMP2 phosphorylation at Thr509 and Thr514.

In our study, we found CRMP2 binds to proteins involved in regulating mitochondrial fission (Drp1) [57] and motility (Miro 2) [58]. Previously, it was shown that CRMP2 interacted with KLC1 [22]. An increased CRMP2 phosphorylation at Thr555 correlated with CRMP2 dissociation from KLC1. In our experiments, we also found interaction of CRMP2 with KLC1 and a negative correlation between CRMP2 phosphorylation at Thr555 and CRMP2–KLC1 interaction. Interestingly, (S)-LCM prevented CRMP2 hyperphosphorylation at Thr509/514 and Ser522 but did not diminish CRMP2 phosphorylation at Thr555 and did not restore CRMP2 interaction with KLC1. Nevertheless, (S)-LCM rescued mitochondrial motility in neurons treated with OA, which caused CRMP2 hyperphosphorylation and CRMP2 dissociation from its binding partners. KLC1 is a protein involved in axonal transport [11] and the restoration of mitochondrial motility with (S)-LCM, paralleled by a decreased CRMP2–KLC1 interaction, seemingly contradicts our hypothesis about CRMP2 involvement in the regulation of mitochondrial traffic. However, earlier it was shown that mitochondrial transport requires kinesin heavy chain and is light chain independent [59]. Consequently, CRMP2 disconnection from KLC1 in our experiments with OA and (S)-LCM does not contradict the restoration of mitochondrial motility produced by (S)-LCM.

CRMP2 hyperphosphorylation in rat cultured cortical neurons incubated in the presence of OA was observed by Lim et al. [50]. In our experiments, we confirmed this observation with mouse cultured striatal neurons. We observed that CRMP2 hyperphosphorylation in the presence of OA disrupted CRMP2 interactions with the proteins involved in regulating mitochondrial dynamics and apparently altered their activity, leading to increased mitochondrial fission and suppressed motility of the organelles. Because phosphatases PP1 and PP2A dephosphorylate CRMP2′s Cdk-5 and GSK-3β sites [20,21], the effects of OA on mitochondrial morphology and motility suggest that CRMP2 phosphorylation at Ser522 by Cdk-5 and at Thr509/514 by GSK-3β are involved in regulating mitochondrial dynamics. Consequently, (S)-LCM could antagonize the effects of OA on mitochondrial dynamics by preventing CRMP2 phosphorylation at these sites. Importantly, genetic deletion of CRMP2 produced very similar alterations in mitochondrial dynamics as OA treatment, suggesting that the changes in CRMP2 expression/phosphorylation may play a key role in regulating mitochondrial morphology.

Okadaic acid might also affect the phosphorylation state of proteins directly involved in regulating mitochondrial dynamics. For example, Cdk-5 and GSK-3β phosphorylate Drp1 [60,61,62], and inhibiting PP1 and PP2A by OA might lead to changes in Drp1 phosphorylation and subsequent alterations in mitochondrial morphology. (S)-LCM prevents hyperphosphorylation of CRMP2 at Thr 509/514 and Ser 522 but does not prevent Drp1 phosphorylation at Ser 616. This suggests that OA-induced alterations in mitochondrial morphology and motility are linked to hyperphosphorylation of CRMP2 but not to Drp1 hyperphosphorylation at Ser 616. Thus, the rescue of mitochondrial morphology with (S)-LCM, which was paralleled by the lack of an (S)-LCM effect on Drp1 phosphorylation, argues against the role of phosphorylated Drp1 in the observed alterations in mitochondrial dynamics.

In our study, the protective effect of (S)-LCM on mitochondrial morphology and motility correlated with prevention of CRMP2 hyperphosphorylation at Thr509/514 and Ser522 but not at Thr555. Subsequently, the protective effect of (S)-LCM correlated with prevention of CRMP2 dissociation from Drp1 and Miro 2 but not from KLC1. These findings suggest that Thr509/514 and Ser522 of CRMP2 and CRMP2 interaction with Drp1 and Miro 2 play the major role in regulating mitochondrial morphology and motility by CRMP2. It is possible that CRMP2 interaction with Drp1 diminishes Drp1 activity and, thus, prevents excessive fission. On the other hand, CRMP2 interaction with Miro 2, an adaptor protein involved in mitochondrial transport [1], could facilitate mitochondrial motility. As a result, dissociation of hyperphosphorylated CRMP2 from Drp1 and Miro 2, observed in OA-treated neurons, increased mitochondrial fragmentation and suppressed mitochondrial motility.

The changes in CRMP2 expression/phosphorylation and resulting alterations in mitochondrial morphology and motility might contribute to various neurodegenerations. Previously, OA treatment of human SK-N-SH neuroblastoma cells was shown to cause CRMP2 hyperphosphorylation at sites aberrantly phosphorylated in AD brain [63]. CRMP2 hyperphosphorylation [14,31], increased mitochondrial fission, and decreased mitochondrial motility [32,33] were found in AD mouse models. CRMP2 hyperphosphorylation in HD brains [22] correlates with excessive mitochondrial fission [23,24,25,26] and reduced mitochondrial motility in HD [25,27,28,29,30]. Taken together, these findings strongly suggest a link between the CRMP2 phosphorylation state and alterations in mitochondrial dynamics. Consequently, manipulations aimed at regulating CRMP2 expression/phosphorylation might be a helpful therapeutic approach in treating various neurodegenerative diseases. Bearing in mind the potential role of CRMP2 in regulating mitochondrial dynamics and given the ability of (S)-LCM to normalize mitochondrial morphology and motility under conditions of CRMP2 hyperphosphorylation, it is conceivable that (S)-LCM could be beneficial in treatments of neuropathologies with hyperphosphorylated CRMP2, such as Huntington’s and Alzheimer’s diseases.

## Data Availability

Data is contained within the article or supplementary material.

## Supplementary Materials

The following are available online at https://www.mdpi.com/article/10.3390/cells10102781/s1, Figure S1: Representative images of neuronal-glial co-culture used in our experiments.
