# Supplementary material for: CRMP2 Is Involved in Regulation of Mitochondrial Morphology and Motility in Neurons
Source: Cells. 2021 Oct 17;10(10):2781. doi: 10.3390/cells10102781 (PMC8535169; doi:10.3390/cells10102781)
Supplement: Supplementary file 1 [file cells-10-02781-s001.zip › cells-1418572-supplementary.pdf]

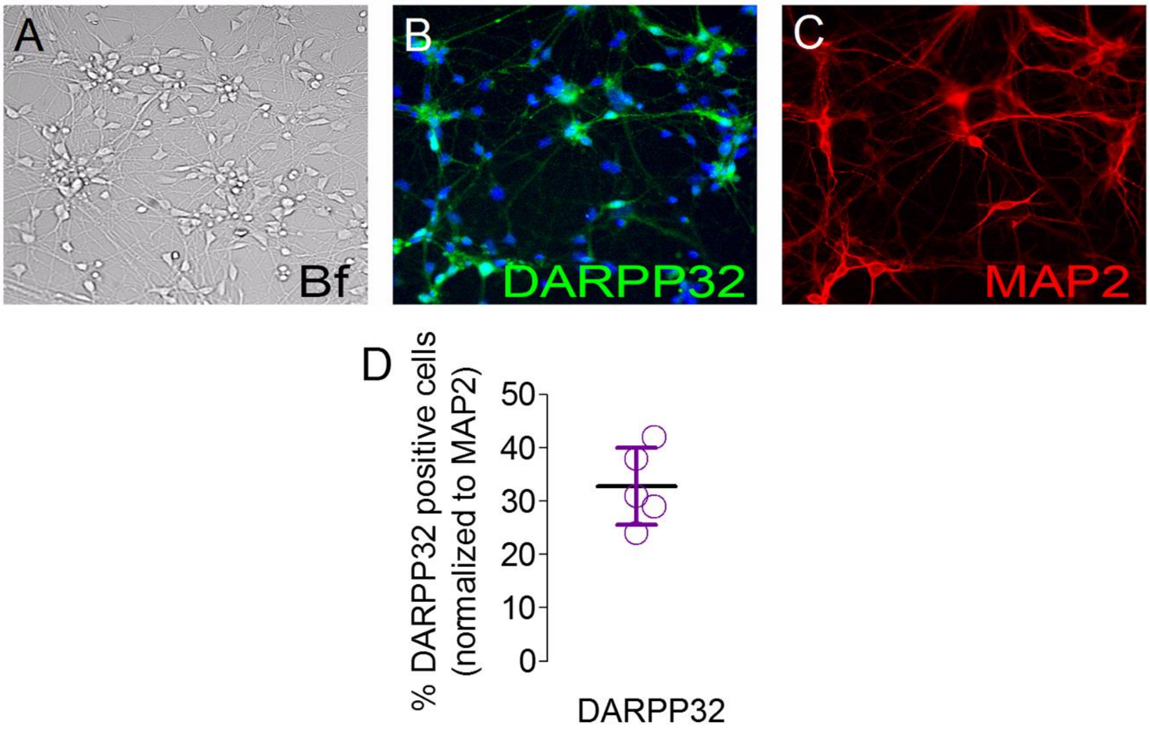

Figure S1. Representative images of neuronal-glial co-culture used in our experiments. (**A**) bright field image. (**B**) DARPP32 staining (green), DAPI, nuclear staining (blue). (**C**), MAP2 staining. (**D**), Percentage of DARPP32-positive cells. Data are mean ± SD, n = 5 separate platings. The colored circles indicate data from individual measurements.
